# New insights into the cellular responses to iron nanoparticles in *Capsicum annuum*

**DOI:** 10.1038/s41598-017-18055-w

**Published:** 2018-02-19

**Authors:** Junxia Yuan, Yu Chen, Huasheng Li, Jinying Lu, Hui Zhao, Min Liu, Galina S. Nechitaylo, Natalia N. Glushchenko

**Affiliations:** 1 Shenzhou Space Biotechnology Group, Beijing, 100190 China; 2 Beijing International Science and Technology Cooperation Base of Space Breeding, Beijing, 100190 China; 3 Emanuel Institute of Biochemical Physics of Russian Academy of Sciences, Moscow, 119334 Russia; 4 V.L. Talrose Institute for Energy Problems of Chemical Physics of Russian Academy of Science, Moscow, 117829 Russia

## Abstract

In this study, the anatomical and ultrastructural responses of *Capsicum annuum* to iron nanoparticles (Fe NPs) were determined. The results showed that the bio-effects of Fe NPs on plants could be positive or negative, depending on the additive concentrations. Low concentrations of Fe NPs were found to promote plant growth. Light and electron microscope analyses showed that the Fe NPs promoted plant growth by altering the leaf organization, and increasing the chloroplast number and grana stacking, as well as regulating the development of vascular bundles. Meanwhile, it was found that the Fe NPs could be absorbed in the roots, and then transported to the central cylinder in bio-available forms, where they were translocated and utilized by the leaves and stems. In contrast, high concentrations of Fe NPs appeared to be harmful to the plants, and the majority of Fe NPs were aggregated into cell walls and transported via the apoplastic pathway in the roots, which may potentially block the transfer of iron nutrients. Taken together, the aforementioned data showed that the rational use of Fe NPs could alleviate iron deficiency, and Fe NPs could be an ideal supply for Fe^2+^ ions fertilizers in agriculture.

## Introduction

Iron (Fe) is a key determinant of the biological functions for a large number of cellular enzymes in organelles, which are important for plant photosynthesis, respiration and for plant product quality^1,2^. The iron element is generally quite abundant in most agricultural environment, however, it has been previously reported that approximately 30% of the world’s soils are iron-limiting for plant growth^3^. Iron deficiency not only negatively affects the growth and development of plants, but also may lead to anemia in animals and humans^4^. Therefore, it is essential to develop an efficient and eco-friendly fertilizer which can improve the efficiency of iron fertilizers in agricultural applications.

Recently, new applications of nanoparticles (NPs) were introduced in the form of nanofertilizers, for the purpose of boosting both crop production and quality. As for iron nanoparticles (Fe NPs), it is one of the aforementioned special nanofertilizers due to its nano size and magnetic characteristics. At first, Fe NPs were widely used for the purpose of remediation of contaminated soil and groundwater. During these processes, nano zero valent iron was oxidized to Fe^2+^ and Fe^3+^ ions, and the organic matter was reduced to inorganic molecular complexes^5^. Meanwhile, the interaction and bio-compatibility between the Fe NPs and the plants attracted a great deal of attentions. According to the records, the Fe NPs have dual effects on plants: low concentrations of Fe NPs were found to have positive effects on the growth and development of plants, whereas high concentrations of Fe NPs seems have resulted in harmful effects on plants^6,7^. Some explanations, which mainly focused on the physiological functions, were proposed. For example, Fe NPs are able to promote nutrient absorption and enhance the photosynthetic efficiency^8–10^. However, the anatomical and ultrastructural responses at the cellular level of plants under Fe NPs exposure remain poorly understood.

When Fe NPs are absorbed by plants, the absorption, distribution, and accumulation of the Fe NPs which are used as fertilizers have also attracted significantly interest in agricultural production. In regard to traditional Fe fertilizers, the iron element is presented in a ferrous form, and plants have developed efficient strategies to acquire iron from their environments. Roschzttardtz *et al*.^11^ performed systematic analyses of the distribution and localization of Fe ions in the model plant *Arabidopsis thaliana*. As stated by Roschzttardtz *et al*.^11^, this “iron map” was an important reference for the research of iron movements within plant tissues. In contrast, the fate of the Fe NPs in plants is not fully understood, and to date, there has been no consensus on this issue. A number of investigations have been reported that the Fe NPs will be able to and unable to be absorbed by plants, depending on the size of nanoparticles^12–14^. Following the absorption process, the fate and transport of the Fe NPs also remain unclear. Some literatures reported that the Fe NPs can be internalized, transported and utilized in vascular tissues, stems, and leaves^15,16^. Other studies held the opposite opinions, such as the particle aggregations of Fe NPs make them too large to be transported within the xylem tissues^17,18^. Therefore, a clear and effective understanding of the absorption, distribution, and accumulation of the Fe NPs is important to illustrate the interactions between Fe NPs and plants, and the answers may potentially constitute a movement framework of the Fe NPs within plants for future studies.

In the present study, the crop plant *Capsicum annuum* was selected for the examinations. Then, the new type of iron fertilizer (Fe NPs) was introduced. There were three specific purposes for this study: (i) The plant growth, along with the anatomical and ultrastructure changes of the *C*. *annuum* elicited by two different concentrations of Fe NPs; (ii) The absorption, distribution and accumulation of the Fe NPs in *C*. *annuum*. (iii) The differences in the performances of the two different concentrations of Fe NPs in *C*. *annuum*.

## Results

### Characterization of the Fe NPs

**Figure 1:**
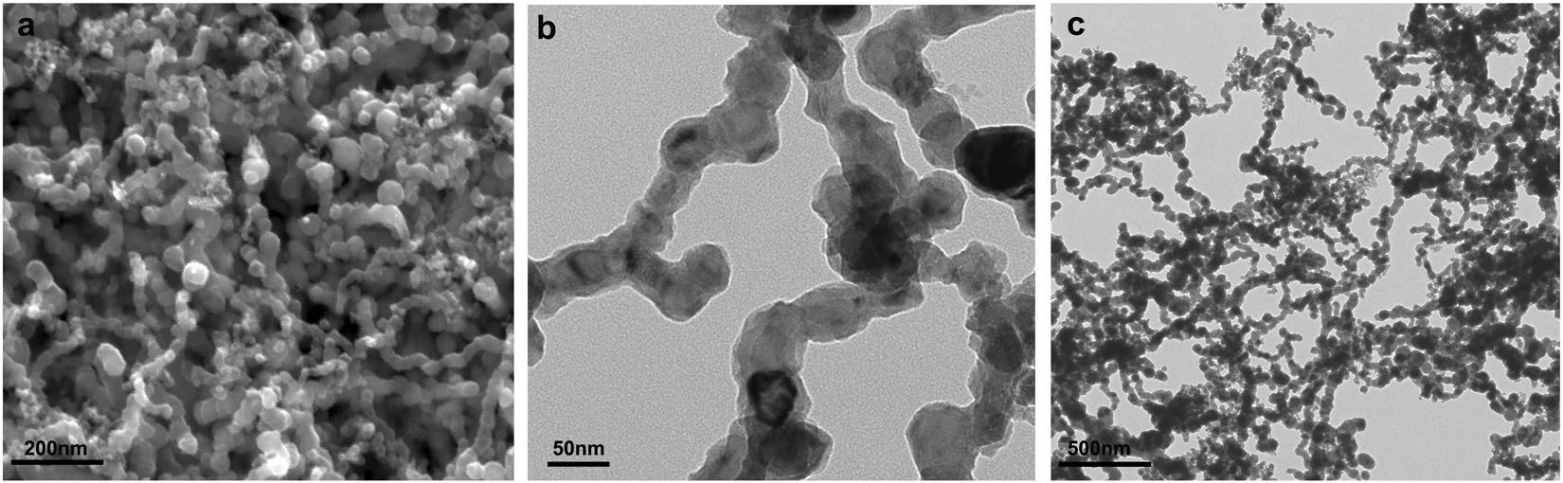
Images of the Fe NPs (**a**, SEM; **b** & **c**, TEM).

In this study, the TEM and SEM images of the Fe NPs showed that a single particle was a rounded shape, and the size was verified to be 52.4 nm ± 5.1 nm. The Fe NPs appeared to be agglomerated forming chain-like formations under laboratorial conditions (Fig. 1). The hydrodynamic diameters of the Fe NPs were approximately 143.8 nm ± 90 nm, and the zeta potentials of the Fe NPs were determined to be negative at −23.3 mV ± 1.2 mV.

### Effects of the Fe NPs on plant height

**Figure 2:**
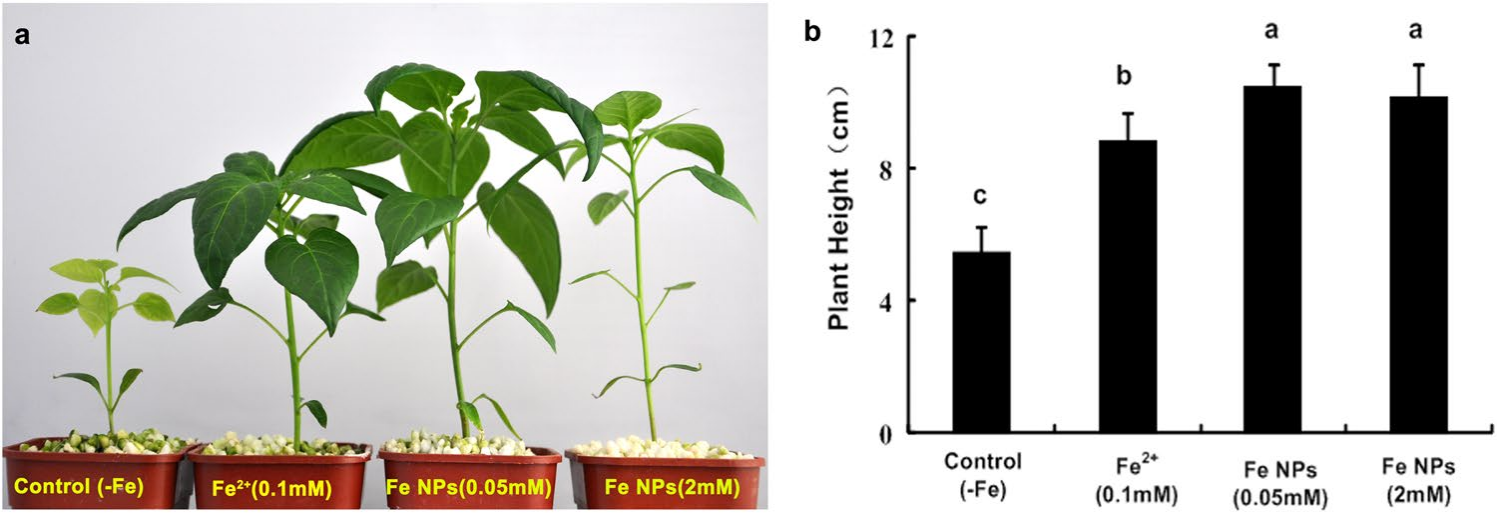
Effect of the Fe NPs on plant height of *C*. *annuum* (Different letters represent significant differences among treatments).

As shown in Fig. 2, the seedlings exhibited green phenotype of the leaves under the Fe^2+^ ions and Fe NPs treatments, whereas, the leaves of the control displayed the typical iron deficiency symptoms as chlorosis. Then, at 35 days after the seeds germination, it was observed that the control produced the lowest plant height, and the 0.05 mM/L Fe NPs promoted the highest plant height, at almost twice that of the control (Fig. 2; p <0.05). The plant height of the 2 mM/L Fe NPs-treated seedlings were lower than that of the 0.05 mM/L Fe NPs treatment, however, the difference was not significant(på 0.05). Therefore, the result clearly indicated that the Fe NPs had significant influences on plant height when compared with the control and Fe^2+^ ions treatment.

### Microscopy characterization

**Figure 3:**
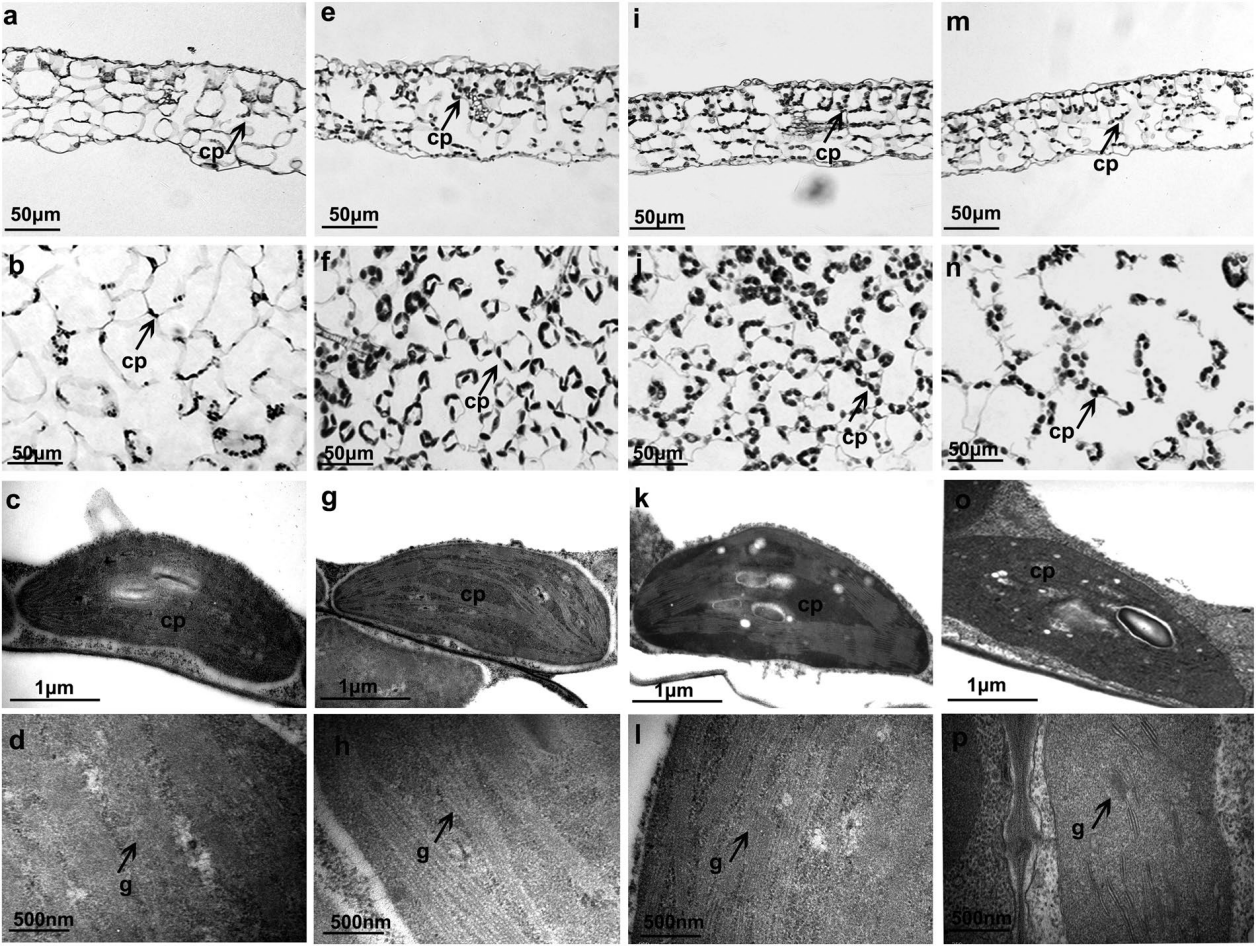
Effects of different iron sources on anatomy and ultrastructure of the leaves in *C*. *annuum* (**a**–**d** control; **e**–**h** Fe^2+^ treatment; **i**–**l** 0.05 mM/L Fe NPs treatment; (**m–p**) 2 mM/L Fe NPs treatment). Abbreviation: chloroplast (cp), grana (**g**).

**Figure 4:**
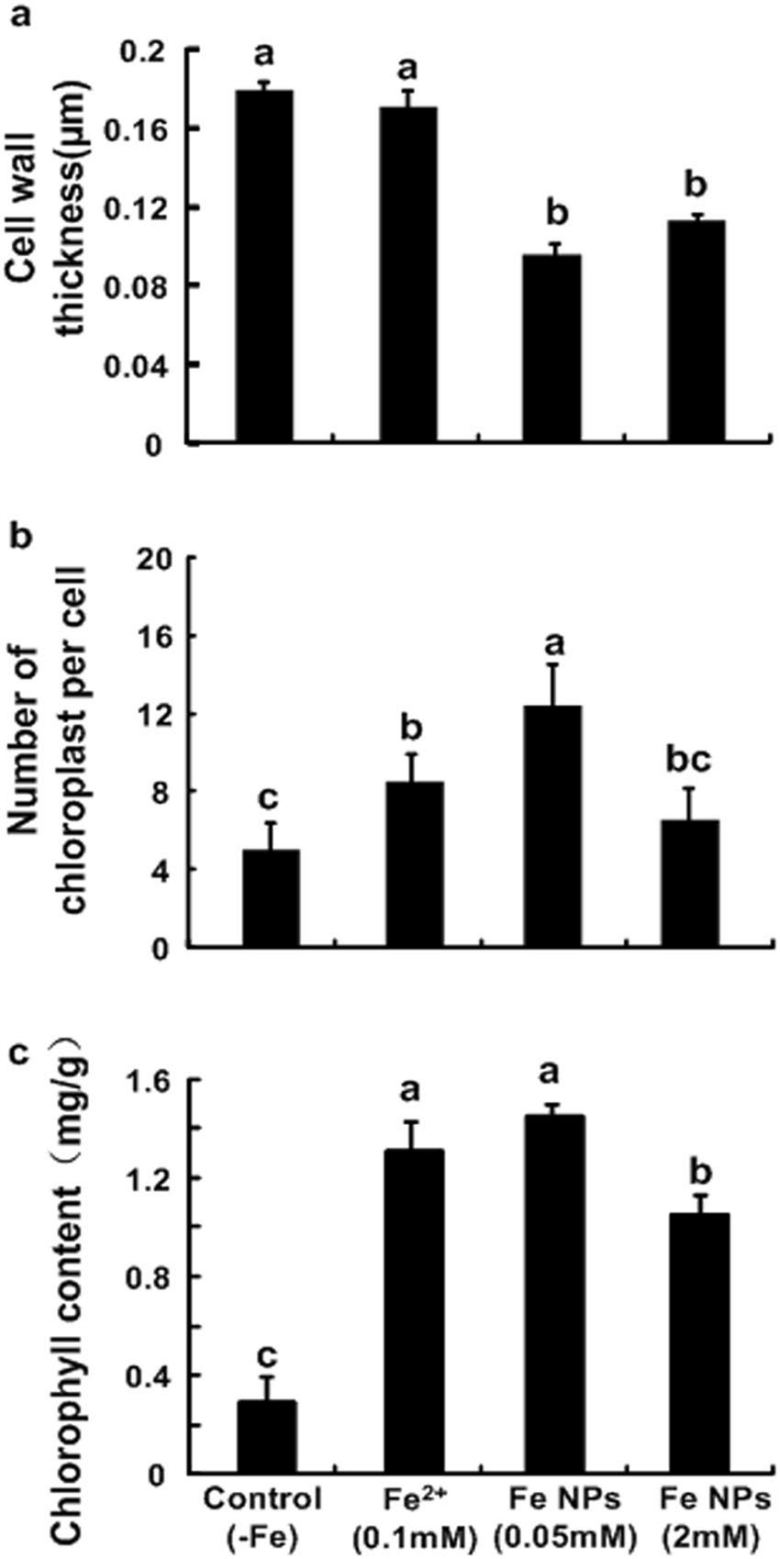
Effects of different iron sources on cell wall thickness (**a**), chloroplast numbers (**b**) and chlorophyll content (**c**) in *C*. *annuum* (Different letters represent significant differences among treatments).

Figure 3a,b showed the anatomical features of the leaves in control plant. The leaves exhibited loosely packed mesophyll cells, and few chloroplasts per cell. With the addition of the iron element, the anatomical features were observed to change significantly. Meanwhile, the leaves displayed different characteristics under the Fe^2+^ ions and Fe NPs treatments. Under the 0.05 mM/L Fe NPs treatment, the leaves exhibited more tightly packed mesophyll cells, and the leaves were observed to become thinner than those of the Fe^2+^ ions treatment (Fig. 3e,i; Supplementary Table A). Similar changes were also observed in the 2 mM/L Fe NPs treated leaves (Fig. 3m; Supplementary Table A). Furthermore, with the Fe NPs treatment, the cell wall of the mesophyll cells were found to become looser, rougher and thinner than those of the control and Fe^2+^ ions treatments (p < 0.05) (Fig. 4a).

Moreover, the anatomical features of the vascular tissues of leaf veins and stems were determined to have changed under the different treatments. For example, in the stems and leaves, the vascular tissue is normally composed of many discrete vascular bundles. However, due to the iron deficiency, the vascular tissues were observed to be severely damaged in the control (Supplementary Fig. 1a,b). Compared to Fe^2+^ ions treatment, the 0.05 mM/L Fe NPs treated seedlings exhibited more tightly packed vascular bundles, while the numbers of vascular bundles was decreased under the 2 mM/L Fe NPs treatment (Supplementary Fig. 1), suggesting that the Fe NPs may potentially affect the organization of the vascular bundles.

To identify the effects of the Fe NPs on the chloroplast, this study examined the chloroplast number per mesophyll cell in the mature leaves of the plants. The control group was found to have produced the lowest chloroplast number per mesophyll cell, as shown in Fig. 4b. While, an increased chloroplast number per mesophyll cell (42.2% increase compared to the Fe^2+^ ions treatment, p < 0.05) was observed under the 0.05 mM/L Fe NPs treatment (Figs 3f,j and 4b). In contrast, a decreased chloroplast number per cell (51.1% decrease compared to the 0.05 mM/L Fe NPs treatment, p < 0.05) was observed under the 2 mM/L Fe NPs treatment (Figs 3n and 4b), suggesting that low concentrations of Fe NPs could be attributed to the increased chloroplast number per mesophyll cell.

This study further examined the ultrastructure of the chloroplast of the mesophyll cells. Due to the iron deficiency, the chloroplast envelope and grana were severely disrupted in the control (Fig. 3c,d), while the ultrastructure of the chloroplast was found to be in typical form, with well-organized grana, lamellar network and well development outer membrane under the Fe^2+^ ions treatment (Fig. 3g,h). Under the 0.05 mM/L Fe NPs treatment, the grana contained relatively more lamellae than that of the Fe^2+^ ions treatment (Fig. 3k,l). However, a significant grana lamellar disintegration was observed under the 2 mM/L Fe NPs treatment (Fig. 3o,p), indicating the chloroplast ultrastructure could potentially be altered by the different forms and concentrations of the iron element.

### Chlorophyll content of the *C*. *annuum*

The results showed that the control group produced the lowest levels of chlorophyll content. Compared with the control, the chlorophyll content were found to be significantly increased in the plants grown with the Fe^2+^ ions and 0.05 mM/L Fe NPs treatment (p < 0.05) (Fig. 4c). Also, significantly decreased values of chlorophyll content were observed in the plants grown with the 2 mM/L Fe NPs treatment (Fig. 4c).

### Distribution of the Fe NPs in the *C*. *annuum*

**Figure 5:**
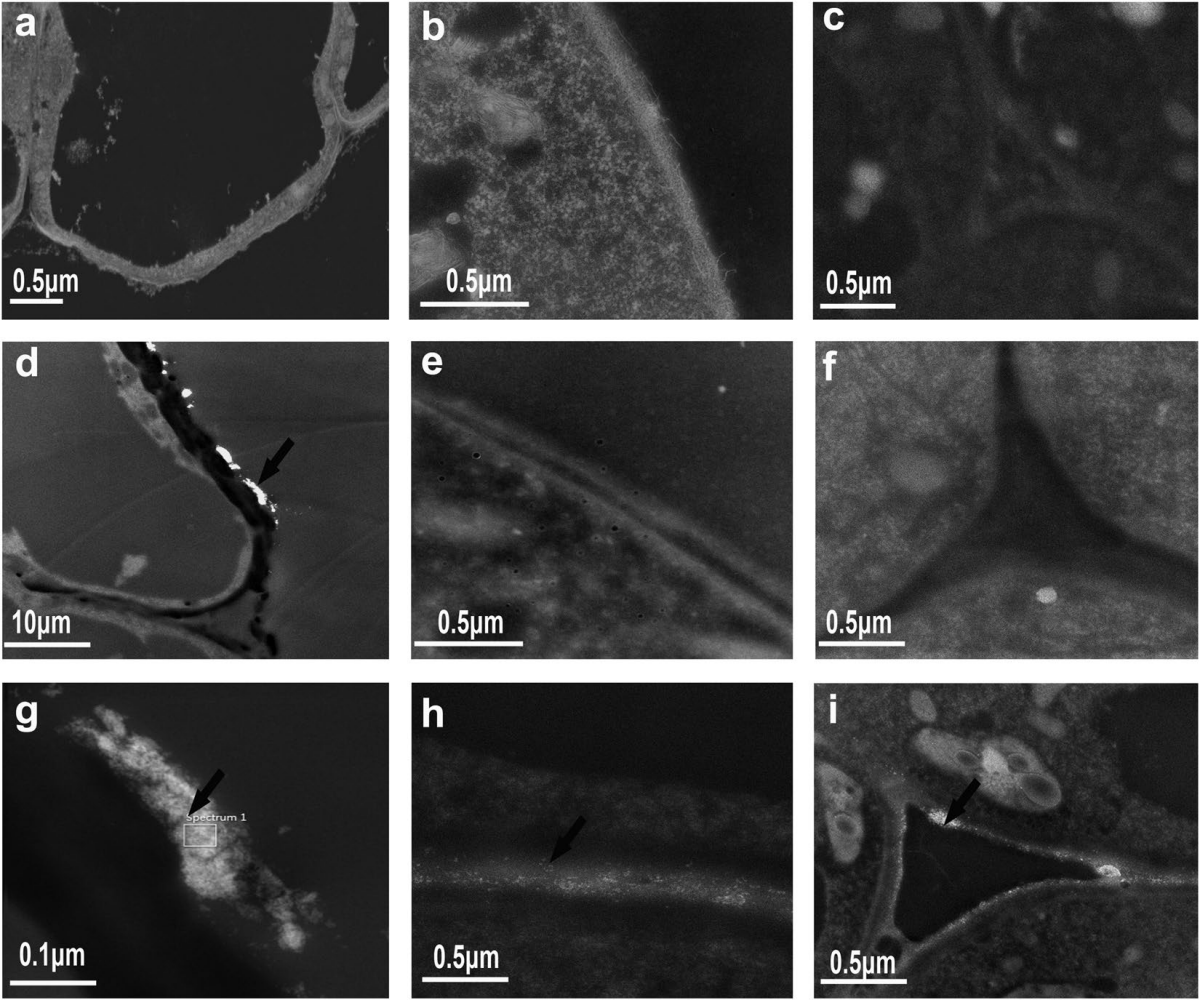
TEM images of epidermis (**a**,**d**,**g**), cortex (**b**,**e**,**h**) and endodermis cells (**c**,**f**,**i**) of plant root treated with the Fe NPs (**a**–**c** Fe^2+^ treatment; **d**–**f** 0.05 mM/L Fe NPs treatment; **g**–**i** 2 mM/L Fe NPs treatment). The arrow pointed to the Fe NPs particles.

In order to more precisely visualize the absorption and distribution of the Fe NPs in the roots, the roots sections of the epidermis, cortex, endodermis and central cylinder were examined. As was expected, there were no visible particles of iron in the root sections under the Fe^2+^ ions treatment (Fig. 5a,b,c). Compared to the Fe^2+^ ions treatment, the distribution of the Fe NPs in the roots was found to display different patterns in terms of the different concentrations. For Example, in the 0.05 mM/L Fe NPs treatment, only a few particles were observed in several outer layers of the epidermis cells (Fig. 5d), while the Fe NPs particles were not found along the arrangement of the cortex, endodermis and central cylinder (Fig. 5e,f). In contrast, majority of the Fe NPs were observed to be aggregated on the epidermis cells, had penetrated the extracellular space, and had reached the endodermis and central cylinder under the 2 mM/L Fe NPs treatments (Fig. 5g–i). It should be noted that the Fe NPs particles were only found to be aggregated on the cell wall, instead of within the cytoplasm (Fig. 5e,f,h,i), which indicated that the Fe NPs had moved by the appoplastic pathway in the roots. This study further examined the distribution of the Fe NPs in the aboveground sections of the *C*. *annuum*, and the results showed that the Fe NPs had not been transported into the stems and leaves via the vascular tissues.

**Figure 6:**
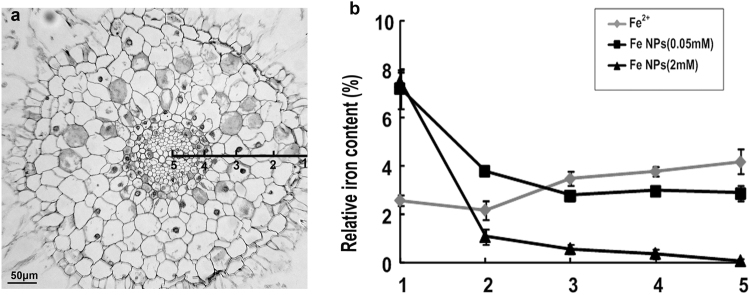
Relative iron content at the sub-cellular level in the roots under different iron treatments (**a**: transverse section of root tip; **b**: the relative iron content in sub-celluar of root).

At this point, the relative iron content at the sub-cellular level in the roots was analyzed by an EDS analysis. The arrangement of the root cells from the epidermis to the central cylinder was marked as Site1 to 5 (Fig. 6a). For the Fe^2+^ ions treated roots, the relative iron content within the cytoplasm were found to be relatively stable among the epidermis, cortex, and endodermis. The highest iron content was detected in the central cylinders (Fig. 6b). Under the Fe NPs treatment, the relative iron content in the epidermis cells was highest, while the relative iron content within the cytoplasm was observed to be stabilized after a sharp decrease, which began in the cortex in 0.05 mM/L Fe NPs treatments (Fig. 6b). Under the 2 mM/L Fe NPs treatment, the relative iron content within the cytoplasm were found to be decreased, with the highest level in the epidermis cells to an extremely low level in the central cylinder cells, suggesting that the iron absorption in the roots had been blocked.

### Total iron content in the *C*. *annuum* plants

**Figure 7:**
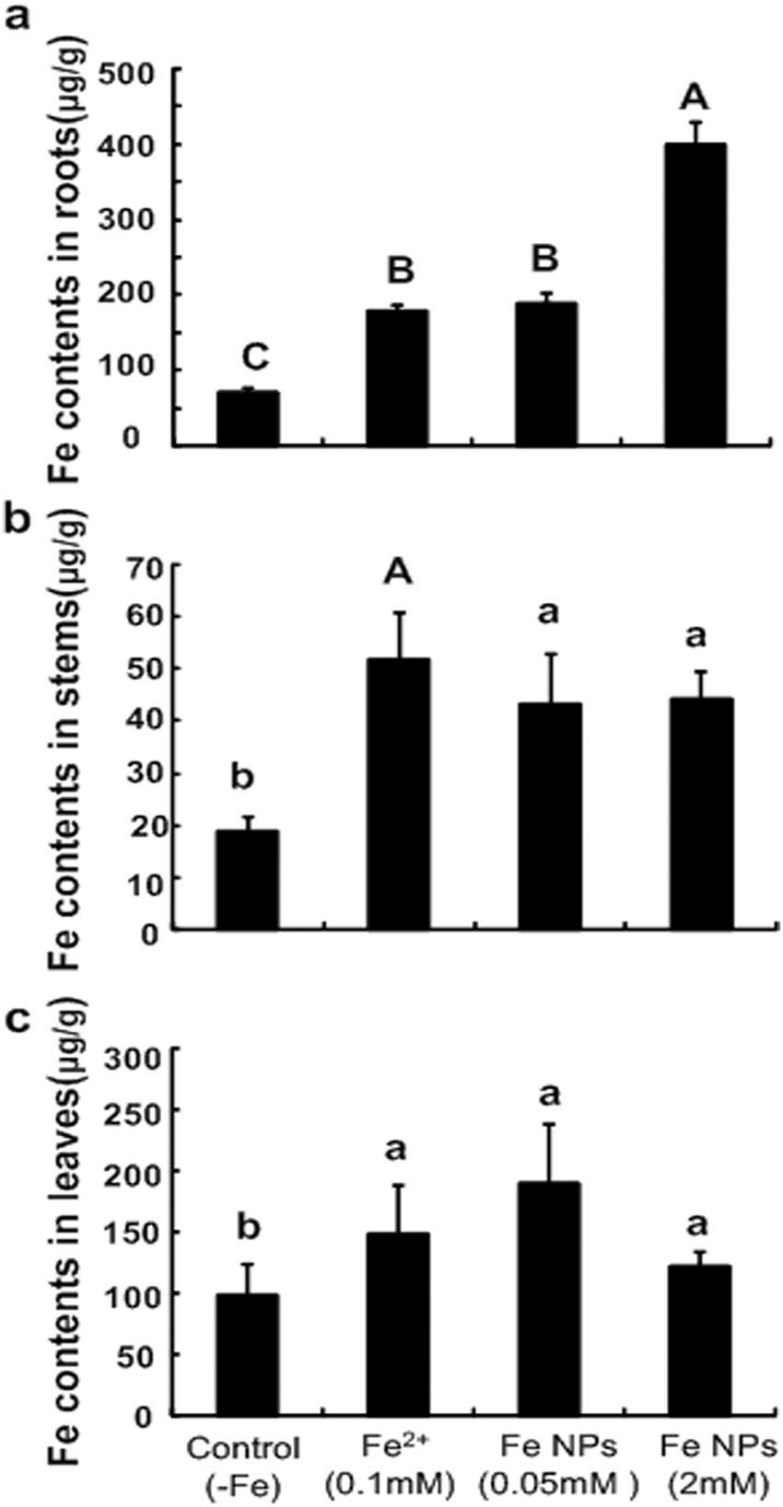
Total iron content in the roots, stems and leaves of *C*. *annuum* plant under different iron treatments. (Different letters represent significant differences among treatments).

As can be seen in Fig. 7, the control group produced the lowest iron content in the roots. Compared to the control, the total iron contents in the roots were significantly increased under the Fe^2+^ ions and 0.05 mM/L Fe NPs treatments. The highest iron content in the roots was found in the 2 mM/L Fe NPs treatment (Fig. 7a).

In regard to the stems, it was also observed that increases in the iron content were evident in the Fe^2+^ ions and Fe NPs treatments, compared to the control (Fig. 7b). Also, there was no significant difference observed between the Fe^2+^ ions and Fe NPs treatments (p > 0.05). In leaves, there were similar patterns of iron content changes among the control and other iron treatments (Fig. 7c), suggesting that the utilizations of the iron were equally between the Fe NPs and Fe^2+^ ions treatments.

## Discussion

In this study, the biological effects of Fe NPs, along with its applicability to promote plant growth, were evaluated. Based on the results, it was determined that the effects of Fe NPs on plants could be either positive or negative, depending on the additive concentrations. Low concentrations of Fe NPs had positive effects on plant growth, whereas, high concentrations of Fe NPs seemed to be adverse for the plants in this study (Fig. 2). According to the literature published to date, this result was consistent with previously studies, and there is consensus that plants responses to Fe NPs are does dependent.

To date, the use of Fe-based nanomaterials in plant fertilization to optimize agricultural production is urgently needed. Lots of studies have shown the results of the promotion of Fe-based nanomaterials in plant production. For example, Sheykhbaglou *et al*.^19^ found that Fe_2_O_3_ NPs were able to increase the leaf and pod dry weights in soybean crops. Trujillo-Reyes *et al*.^20^ reported that the Fe_3_O_4_ NPs had the ability to increase the antioxidant enzyme activities of lettuce. Also, Rui *et al*.^21^ compared the effects of Fe_2_O_3_ NPs and Fe-EDTA on the growth of peanut, and the results showed that the Fe_2_O_3_ NPs promoted increased root lengths, plant height, biomass and SPAD values of peanut. However, these observed physiological effects are far from explaining the cellular mechanisms for Fe-based nanomaterials in plants.

At the cellular level, the possible mechanisms for the promotional effects of Fe NPs mainly include the structure and functions changes of the biological components. In this study, a systematic analysis was performed regarding the effects of the Fe NPs on the cellular changes in *C*. *annuum*. It was speculated that the Fe NPs may have potentially regulated the plant growth based on the following evidence. First of all, the low concentrations of Fe NPs played a positive role in the formation of more chloroplasts, as well as more tightly packed chloroplast grana, when compared with the control and Fe^2+^ ions treatment (Figs 3 and 4a). The chloroplast biogenesis and grana organization are known to be two vital events of chloroplast development, and are important for the light capture process during photosynthesis^22^. Also, it has been suggested that grana stacking plays an important role in protecting photo system II (PSII; located in the stacked grana), as presented by Anderson and Aro^23^ in 1994. The increased chlorophyll content also suggested the promotion of photosynthesis activities in the plant cells (Fig. 4c). This study inferred that the changes in the chloroplasts numbers and structure may increase the photosynthesis efficiency. Secondly, the changes in the cell walls may potentially be beneficial to the rapid elongations of the plants. It is known that, plant cell walls display extreme tensile strength and extensibility, and the changes of mechanical properties in cell walls can not only significantly affect the pore size distribution and permeability of cell wall but also the expansion of the cells^24^. Recently, Kim *et al*.^7^ provided a new insight in which Fe NPs oxidation can lead to the production of OH radicals, which can trigger cell wall loosening, facilitate the release of tensional stress in cells, and finally enhance the elongation of plants. Thirdly, the vascular bundles were observed to be increased by the low concentrations of Fe NPs, and it was predictable that the transport of nutrients were enhanced coordinating with more development structures.

As the Fe NPs concentration increased to 2 mM/L, the deformed and damaged vascular bundles were observed in the *C*. *annuum*. A similar finding was published in previous study, in which high concentrations of nanomaterials could potentially alter the absorption of nutrients in cottons^25^.

Moreover, as the goal of this study is to discuss the applicability of Fe NPs as a fertilizer, the fate and transport of Fe NPs and Fe^2+^ ions fertilizers were comparatively studied, and it was concluded that the fate of iron elements were associated with the form and additive concentrations of the iron. For the Fe^2+^ ions, plants have developed a strict regulation of iron absorption, long-distance transport, storage, and remobilization^26^. Roschzttardtz *et al*.^11^ confirmed that plant roots mainly accumulate iron in the apoplast of the central cylinder. Then the Fe^2+^ ions can be transported to the shoots through the xylem tissues.

Unlike Fe^2+^ ions, the Fe NPs are present in an insoluble form, therefore, the method by which the Fe NPs behave inside the roots was worthy of studying. Under the low concentrations of Fe NPs treatment, few particles were only observed in several outer layers of the epidermis cells, and the absence of visible particles within the cytoplasm suggested that the Fe NPs were not transported across the plasma membranes. Meanwhile, it was interesting to note that the relative iron content within the cylinder cells was close to the level of the Fe^2+^ ions group (Fig. 6). All of the accumulated evidence indicated that the insoluble Fe NPs may be transformed into bio-available forms (for example, Fe^2+^ or Fe^3+^) and then transported toward the vascular tissues to the stems and leaves (Figs 6 and 7). These findings were found to be congruent with the results of Keller *et al*.^27^, in which the Fe NPs applications were substantially increase the concentrations of Fe^2+^ and/or Fe^3+^. Therefore, when combined with the beneficial changes in the plants, such as increased chloroplast, tightly packed grana, and increased chlorophyll content under the low concentrations of Fe NPs, this study considered that the addition of low concentrations of Fe NPs could be absorbed and utilized by plants, and Fe NPs may potentially be an ideal supply for Fe^2+^ ions fertilizers.

Then, how the Fe NPs permeated into the root is another interesting question. Results of the Fe NPs distribution in the roots under the high concentrations treatment provided evidence that the Fe NPs had moved by the appoplastic pathway in the roots (Fig. 5). The apoplastic location of the Fe NPs was supported by previous viewpoints that the nanoparticles larger than 20 nm are unable to penetrate through the cell walls^28,29^. Therefore, to support the plant growth, the insoluble Fe NPs should be absorbed in the form of Fe^2+^ or Fe^3+^ under both the low and high concentration of Fe NPs treatment. Unlike the low concentration of Fe NPs treatment, the clogging effects of Fe NPs in apoplast under the high concentration treatment may lead to extremely low levels of iron content in the central cylinder cells (Fig. 6), and may finally severely block the nutrients transport (e.g. iron). This view point was supported by numerous studies that the potential adherence to the root causes adverse effects in plants when the concentration is relatively high^27,30,31^. Also, the iron deficiency in the central cylinder cells may lead to structural damages in the plants. These damages were mainly reflected in the deformed chloroplast, damaged vascular bundles, decreased chlorophyll content, and so on (Figs 3 and 4c; Supplementary Fig. 1). However, it should be pointed out that the reactive oxygen species (ROS) which is produced by Fe NPs oxidation may be another factor which is harmful to plants^32,33^. Therefore, improvements in the understanding of the toxicological effects of Fe NPs will also be required in further studies.

## Conclusion

This study found that the biological effects of iron nanoparticles on plants can be positive or negative, depending on the additive concentrations. Low concentrations of Fe NPs promoted plant growth at the cellular level by altering the leaf organization, increasing the chloroplast number and grana stacking, and regulating the development of vascular bundles. Also, there was no bioaccumulation of Fe NPs in the plant tissues, and Fe NPs may potentially be an ideal supply for Fe^2+^ ions fertilizers.

## Methods

### Characterization of the Fe NPs

The Fe NPs were provided by the Russian Academy of Sciences (RAS). The size and morphology of the Fe NPs were characterized using transmission electron microscopy (JEM-100CX II) and scanning electron microscopy (S250MK3). The hydrodynamic size of particles and zeta potentials were analyzed by dynamic light scattering (DLS) using a particle size analyzer (Zetasizer Nano ZS).

### Growth of the plants

The *Capsicum annuum* were grown on horticultural perlite in a green house at 26 °C. The seeds were sown and irrigated with deionized water. Following germination, the seedlings were transferred into different plant pots and irrigated with MS medium^34^ with slight modifications as follows. In the preliminary test, the MS medium without any iron content was used as the control, and 0.002–2 mM/L of Fe NPs and Fe^2+^ ions were designed, and 0.05 mM/L and 2 mM/L of Fe NPs in further experiments were selected because the most significant difference was observed between the two treatments. Meanwhile, 0.1 mM/L of Fe^2+^ ions was used in further experiments that can meet the demand of plant growth. In this study, four different treatments of iron element were designed: (1) the MS medium without any iron content was used as the control; (2) the standard MS medium containing 0.1 mM/L of Fe^2+^ ions; (3) the MS medium (without Fe^2+^ ions) containing 0.05 mM/L of Fe NPs; (4) the MS medium (without Fe^2+^ ions) containing 2 mM/L of Fe NPs. The suspensions of Fe NPs were prepared in deionized water, and dispersed by an ultrasonic bath (Scientz-IID) for 15 minutes. Then, they were subsequently added to the MS medium nutrient solution. In the experiment, the seedlings were watered every three days using different above-mentioned nutrient solutions respectively.

### Microscopy

The root, leaf and stem samples from the control and the iron treatments were examined by light and electron microscopy at the end of the 20 days growth period.

For light microscopy, the samples were fixed in FAA solution under a vacuum, overnight at room temperature. Then, the samples were dehydrated in an ethanol series and dimethylbenzene series, embedded in paraffin, sectioned using a rotary microtome at 2 μm and stained with safranine-fast green and finally mounted. The sections were observed and photographed using Olympus BX41 light microscopy.

For transmission electron microscopy, the samples were respectively fixed, embedded and dehydrated as described by Yuan and Xu^35^. Samples were fixed in glutaraldehyde (2% in a 0.074 M sodium cacodylate buffer) and rinsed twice in the buffer. All steps were performed at room temperature. The samples were dehydrated twice in an ethanol series and 100% acetone washing and finally embedded in epon resin. Approximately 100 nm ultra-thin sections were prepared on a Leica ultramicrotome (EMUC6). One half of the ultra-thin sections were analyzed using the high resolution transmission electron microscope (JEM-2100F) and energy dispersive spectroscopy (EDS) to reveal the absorption and distribution of iron elements, and the other half of sections were stained with a saturated solution of uranyl acetate and lead citrate and photographed with a Hitachi H-7650 transmission electron microscope to analyze the ultrastructure features of plants.

### Plant height, chlorophyll and iron content determination

After 35 days from seeds germination, the plant height was measured. The fresh leaves were harvested, and leaf chlorophyll content was determined according to Lichtenthaler and Wellburn^36^. As for iron content determination, the harvested *C*. *annuum* plants were separated in to roots, stems and leaves. All the fresh plant tissues were dried in a 105 °C chamber for 72 h, and 150 mg of dry samples were taken after incubation to measure the iron content using inductively coupled plasma optical emissions pectrometry (ICP-OES; ICAP6300) as described by Rui *et al*.^21^. The leaf chlorophyll content and iron content measurements were based on three independent replicates, separately.

### Statistical analyses

In this study, each exposure was made ≥3 replicates. The results were given as means ± standard error. The significance of the influence of iron on plant morphological and cellular characteristics was tested by ANOVA at a significance level of 0.05.

## Electronic supplementary material

Supplementary information
