# Supplementary material for: New insights into the cellular responses to iron nanoparticles in Capsicum annuum
Source: Sci Rep. 2018 Feb 19;8:3228. doi: 10.1038/s41598-017-18055-w (PMC5818496; doi:10.1038/s41598-017-18055-w)
Supplement: Supplementary file 1 — Supplementary information [file 41598_2017_18055_MOESM1_ESM.pdf]

New insights into the cellular responses to iron nanoparticles in *Capsicum annuum*

Junxia Yuan[1, 2] Yu Chen[1, 2] Huasheng Li[1, 2] Jinying Lu[1, 2*] Hui Zhao[1, 2*]
Min Liu[1] Galina S. Nechitaylo[3] Natalia N.Glushchenko[4]

[1]Shenzhou Space Biotechnology Group, Beijing 100190, China
[2]Beijing International Science and Technology Cooperation Base of Space Breeding, Beijing 100190, China
[3] Emanuel Institute of Biochemical Physics of Russian Academy of Sciences, Moscow 119334, Russia
[4] V.L. Talrose Institute for Energy Problems of Chemical Physics of Russian Academy of Science, Moscow 117829, Russia

*Corresponding authors:

Jinying Lu

Address: No. 61 Zhichun Road Haidian District, Beijing 100190, China

E-mail: lujinying2001@sina.com

Fax: 86-10-68378293

Phone: 86-10-68379018

Hui Zhao

Address: No. 31 Zhongguancun South Street Haidian District, Beijing 100080, China

E-mail: zhaohui@china.com

Fax: 86-10-68378293

Phone: 86-10-68196688

Supplementary Fig. 1

Anatomical changes of the vascular tissue of leaf veins (a,c, e, g) and stems (b, d, f, h) under different iron treatments (a, b control group; c, d Fe^{2+} treatment; e, f 0.05mM/L Fe NPs treatment; g, h 2mM/L Fe NPs treatment).

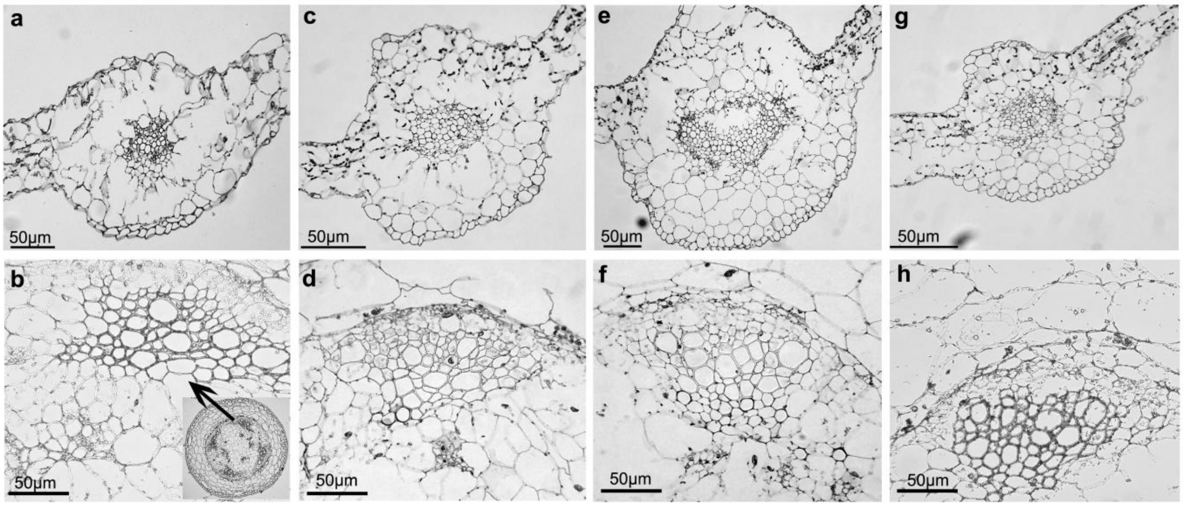

Supplementary Table 1

Table 1 Effects of iron sources on anatomical structure of *C. annuum* leaves

Treatments	Leaf thickness（μm）	Palisade tissue/Spongy tissue
Control	138.02±5.96 a	0.38±0.03 b
Fe^{2+}（0.1Mm/L）	136.73±4.14 a	0.40±0.05 b
Fe NPs （0.05Mm/L）	97.82±3.42 b	0.52±0.06 a
Fe NPs （2Mm/L）	99.65±5.50 b	0.49±0.04 a

*Different letters indicates significant differences among treatments (P＜0.05).
